# Indirect Sensing of Lower Aliphatic Ester Using Atomic Gold Decorated Polyaniline Electrode

**DOI:** 10.3390/s20133640

**Published:** 2020-06-29

**Authors:** Parthojit Chakraborty, Yu-An Chien, Tso-Fu Mark Chang, Masato Sone, Takamichi Nakamoto

**Affiliations:** 1 Department of Information and Communications Engineering, Tokyo Institute of Technology, Kanagawa 226-8503, Japan; chakraborty.p.aa@m.titech.ac.jp; 2 Department of Material Science and Engineering, Tokyo Institute of Technology, Kanagawa 226-8503, Japan; chien.y.aa@m.titech.ac.jp; 3 Institute of Innovative Research (IIR), Tokyo Institute of Technology, Kanagawa 226-8503 Japan; chang.m.aa@m.titech.ac.jp (T.-F.M.C.); sone.m.aa@m.titech.ac.jp (M.S.); 4 Laboratory for Future Interdisciplinary Research of Science and Technology, Institute of Innovative Research, Tokyo Institute of Technology, Kanagawa 226-8503, Japan

**Keywords:** amperometric gas sensor, bi-atomic gold, ethyl formate, polyaniline, principal component analysis

## Abstract

Novel sensing materials have been formed by decorating polyaniline conducting polymers with atomic gold clusters where the number of atoms is precisely defined. Such materials exhibit unique electrocatalytic properties of electrooxidation to aliphatic alcohols, although analytes with other functional groups have not been studied. This paper reports a study of cyclic voltammetric patterns obtained with bi-atomic gold nanocomposite response to analytes with other functional groups for sensor applications. Principal component analysis shows separation among normal-propanol, iso-propanol and ethyl formate/ethanol groups. Indirect sensing of ethyl formate is demonstrated by electrooxidation of the product upon hydrolysis in alkaline medium. Voltammograms of ethyl formate are studied in gaseous phases.

## 1. Introduction

Gas sensing systems are known to have a variety of applications in health, security, energy, etc. Although it is essential to have sensor devices with sufficient sensitivity and selectivity, there is still room for improving the sensor characteristics. Thus, we focus on noble metal-based catalytic materials, i.e., atomic gold. It has been reported that novel sensing materials, such as noble metal nanoclusters [1] and metal oxides [2,3], show interesting catalytic properties. With noble metal nanoclusters, it has been demonstrated that while bulk metal electrode exhibits some catalytic activity, the activity is greatly enhanced when bulk metal is reduced to nanocluster size [4]. For example, alcohols catalyzed by metal clusters have shown electrooxidation of 2-propanol to its corresponding ketone in alkaline medium.

Single-atom catalysts (term first proposed in 2011) have recently shown to be a new frontier due to their high activity and selectivity for various chemical reactions. Over the years, several groups have demonstrated the catalytic effect of metal nanoclusters and single-atom catalysts [5]. However, isolation of single atoms is challenging and a suitable substrate or support matrix is required to make a stable isolated atomic structure. Although so far metal oxides of nickel, zinc, titanium and others have been the most common substrates, conducting polymers such as polyaniline (PANI) have also been recently proven to serve as a suitable support matrix for dispersing metal nanoparticles [6,7]. PANI is an attractive material due to its high conductivity, ease of preparation, room temperature operation and many tunable redox properties [8,9].

**Figure 1:**
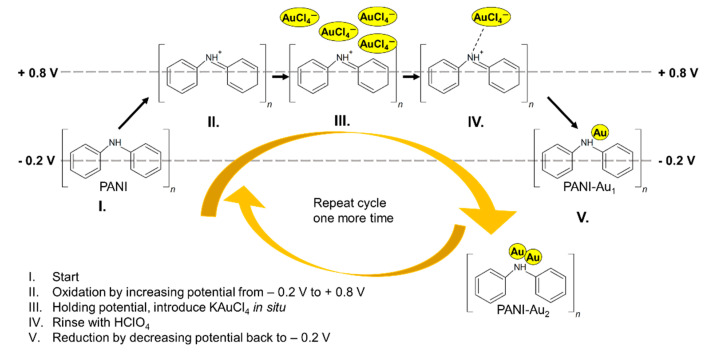
Cyclic pathway of decorating single and bi-atomic gold clusters on the PANI matrix (PANI-Au_1_ and PANI-Au_2_). (I–II) PANI is oxidized to its imine form inside 0.1 M HClO_4_ acidic medium; (II–III) AuCl_4_^−^ ions are introduced into the system with potential held at + 0.8 V; a (III–IV) PANI*AuCl_4_^−^metal halide complex is formed with excess AuCl_4_^−^ ions rinsed away using HClO_4_ buffer solution; (IV–V) AuCl_4_^−^ is reduced to atomic Au.

**Figure 2:**
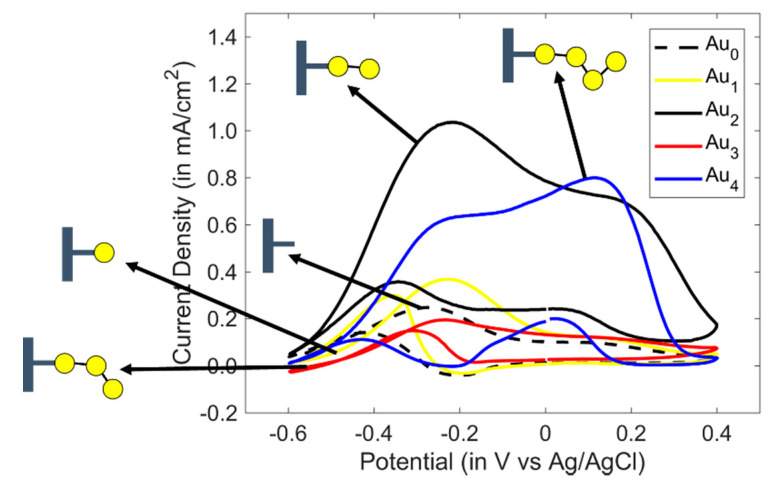
Odd–even pattern in the peak current density due to the HOMO-LUMO energy gap energy variation of atomic size clusters [10,11,12,13,15]; figure shows the electrooxidation of 0.5 M n-propanol in 1 M KOH (scan rate = 100 mV/s).

Although so far, an obstacle to nanocluster growth had been the precisely controlled growth in size of the nanocluster, recent advances have been made with gold (Au), a noble metal, by using PANI in its emeraldine oxidation state as the support matrix (Figure 1). PANI can be and has been electrochemically decorated with atomic size Au through a bottom-up process [10,11]. Cyclic voltammograms (CV) from the electrooxidation of alcohols illustrate an odd–even pattern (Figure 2) due to the variation in the HOMO-LUMO gap energy of the atomic Au cluster [12,13,14,15]. For the electrocatalytic oxidation of n-propanol, the alkoxide ion formed from C-H bond cleavage is actively oxidized to a propionic aldehyde and its intermediates resulting in oxidation peaks. Fourier-transform infrared spectroscopy (FTIR) spectroscopy of N-H stretching measured in the region of 3100–3500 cm^−1^ in 0.1 M HClO_4_ has also been reported [12]. The dependency of N-H stretching shows a similar odd–even pattern obtained from the band areas relative to the number of atoms in the cluster. Since the Au atoms are expected to be close to the nitrogen sites of PANI, the magnitude of N-H stretching vibration therefore depends on the size and stability of those atomic Au clusters in PANI. Features obtained from CV with structured atomic Au-PANI nanocomposites suggest that they can be used as novel sensing materials for sensing applications as well. In this research, PANI electrodes have been decorated with bi-atomic gold and the thus-fabricated nanocomposites have been used as sensing material for the classification between normal-propanol (n-propanol) and iso-propanol (i-propanol). The enhanced sensitivity and selectivity obtained due to the electrocatalytic activity of atomic gold are harnessed as useful properties for sensing in both liquid and gas phases [13,15,16]. Although cyclic voltammetry shows that atomic Au-PANI nanocomposites can be used as sensing material for alcohols, fundamental study needs to be conducted with other functional groups as well. 

This paper reports the electrooxidation of the Pt||PANI-Au_2_ (bi-atomic gold catalyst) polymer nanocomposite to four functional groups viz. ketone, alcohol, carboxylic acid. CV responses followed by principal component analysis are reported. For the ester group, ethyl formate was chosen as it is harmless to the skin and can operate at room temperature as a potential fuel for consumer electronics applications in direct formate fuel cells (DFFCs) [17]. Ethyl formate is also a representative of the ester with its scent, indispensable for food and agricultural products [18]. Indirect sensing of ethyl formate in the gas phase through electrooxidation upon hydrolysis in alkaline medium is reported.

## 2. Materials and Methods

Growing atomic gold clusters atom by atom onto the surface of PANI is a cyclic process. With PANI polymerized at a platinum (Pt) working electrode, linearly sweeping the applied potential from −0.2 V to +0.8 V leads to the electrooxidation of PANI to its imine form. If a metal halide (e.g., tetrachloroaurate anion AuCl_4_^−^) is introduced in the system, the PANI*AuCl_4_^−^ metal complex is formed due to high electron affinity of the PANI functional group in this state. Figure 2 shows the odd–even pattern in the current density for different atomic size clusters. This odd–even alteration due to the number of gold atoms is attributed to the HOMO-LUMO energy gap of the Au cluster, also seen in other research works on Au clusters [19]. Although higher atomic size clusters can be formed, for this fundamental study the simplest structure of bi-atomic Au (PANI-Au_2_) has been selected. PANI-Au_2_ has already shown discriminable CVs among different linear alcohols, which is discussed later. Furthermore, the catalytic effect of the bi-atomic Au-PANI electrode sensor for the gas phase detection of n-propanol has been reported elsewhere [16].

### 2.1. Target Compounds

A list of target compounds is shown in Table 1.

### 2.2. Chemicals and Materials

All chemicals were purchased with purity 98–99.5% from Wako Chemicals Ltd., Osaka, Japan and the Sigma Aldrich Ltd., Tokyo, Japan. Platinum Pt electrode (diameter Ø = 3 mm, BAS Co. Ltd., Tokyo, Japan) was used as the working electrode (WE). Ag/AgCl in 3 M NaCl and Hg/HgO in NaOH (also purchased from BAS Co. Ltd., Tokyo, Japan) were used as reference electrodes (RE) for measurements in acidic medium and alkaline medium electrolytes, respectively. A 0.1 mm Pt thin film (total surface area = 8 cm^2^, Nilaco, Tokyo, Japan) was used as counter electrode (CE).

### 2.3. Synthesis of PANI and PANI-Au_2_ Nanocomposite

For this research, Pt||PANI-Au_2_ (Pt with a PANI-Au_2_ nanocomposite) was used as the working electrode. For nanocomposite preparation, 0.1 M aniline (C_6_H_5_NH_2_) in 2 M tetrafluoroboric acid (HBF_4_) was electropolymerized on the surface of each electrode to prepare uniform PANI film using a three-electrode electrochemical setup. Polymerization of PANI film was successfully confirmed, both visually and electrochemically, from the characteristic redox peaks on the cyclic voltammograms, obtained using 0.1 M perchloric acid (HClO_4_). One of the two electrodes was then chosen for the stoichiometric growth of the bi-atomic Au cluster on the PANI matrix, as illustrated in Figure 1. An amount of 0.2 mM potassium tetrachloroaurate (KAuCl_4_) dissolved in 0.1 M HClO_4_ was used for the electrochemical decoration of atomic gold into the PANI matrix. PANI oxidized to its imine state at +0.8 V formed a PANI*AuCl_4_^−^ metal complex surrounded by excess AuCl_4_^−^ anions. An amount of 0.1 M HClO_4_ was used as a buffer solution for rinsing excess AuCl_4_^−^ anions inside the electrochemical cell for a period of about 800 s. After the rinsing stage, the potential was pulled down to −0.2 V. At this stage, Au^3+^ was reduced to Au^0^. The synthesis of the PANI-Au_2_ nanocomposite was confirmed from the PANI overoxidation peaks after each atomic gold deposition (Figure S1 in Supplementary Material) [11] and from electrooxidation cyclic voltammograms with n-propanol and i-propanol [20]. Stable voltammograms were observed after approximately 30 scans at 100 mV/s. The thus prepared electrode was air-dried and stored in glass tubes under room-temperature conditions.

### 2.4. Instrumentation and Electrochemical Setup

For precise and controlled atomic gold deposition, a polycarbonate-based electrochemical flow cell (Ono Denki, Japan) was designed using 3D CAD software (Fusion 360, Autodesk, California, USA). The configuration of the 3-electrode setup within the flow cell was the same as reported in our previous work [16]. The contact area between the Pt counter electrode and the electrolyte inside the flow cell was 1.21 cm^2^. For gas phase measurements, a single-component gas delivery system, based on our past research, was fabricated [14,21]. The gas delivery system is a dynamic system that uses a headspace sampling method [22] to blend ambient air with the vapor headspace of the target compound. In the system, relative concentration can be specified. In this paper, experiments were limited to a single component and the actual output gas concentration was cross-checked using a photoionization detector (ppbRAE 3000, RAE Systems, California, USA) for the purpose of calibration. A laboratory-fabricated potentiostat, along with other instrumentations, was used to precisely control measurement parameters. Serial-USB protocols inside a MATLAB script file (2017a, Mathworks, Massachusetts, USA) were used to accumulate the data on a PC.

## 3. Results

### 3.1. Study of Cyclic Voltammetry of PANI/Au_2_ with Linear Alcohols

**Figure 3:**
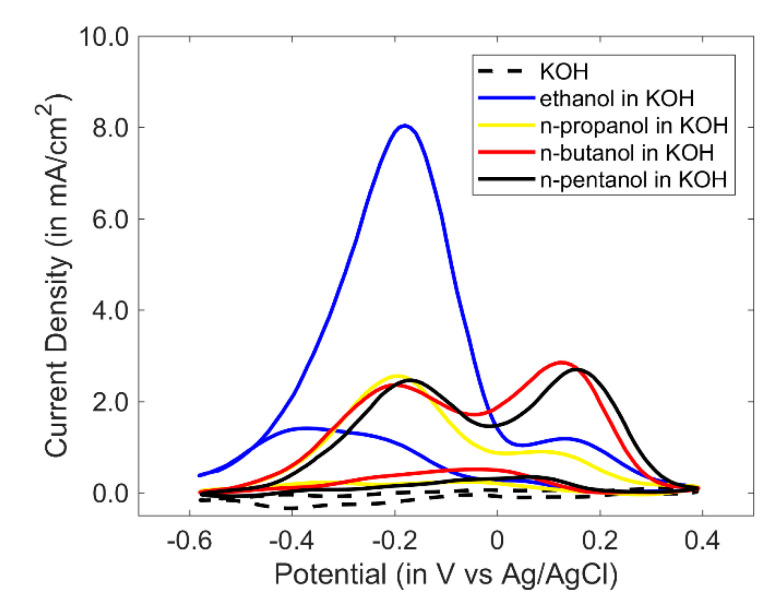
Cyclic voltammograms of PANI-Au_2_ vs. Ag/AgCl for linear alcohols dissolved in 1 M KOH. (Scan rate: 100 mV/s).

Cyclic voltammetry was performed for linear/primary alcohols viz. ethanol, n-propanol, n-butanol and n-pentanol (Figure 3). An amount of 0.5 M of the alcohol was dissolved in 1 M KOH for electrooxidation. The current density at −0.2 V decreased from ethanol to n-propanol. From n-propanol to n-butanol, the current density did not vary much, however there was a remarkable rise in the oxidation peak at +0.2 V for n-butanol.

### 3.2. Survey of Sensor Response across Functional Groups in 1 M KOH Alkaline Medium Electrolyte

**Figure 4:**
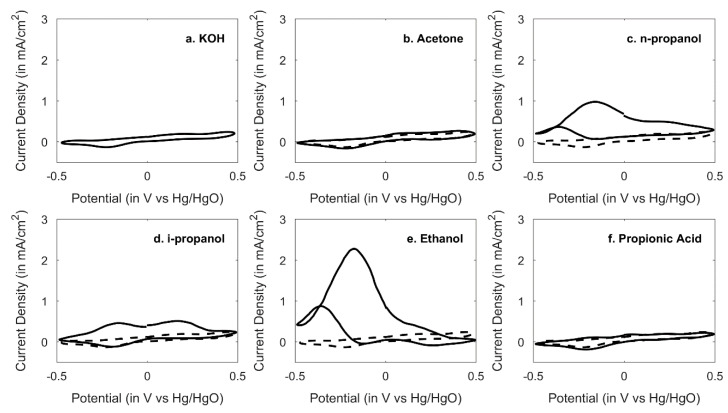
Cyclic voltammograms of Pt||PANI-Au_2_ electrode vs. Hg/HgO for 5 target compounds at scan rate 100 mV/sec. (a = 1 M KOH, b = 0.5 M acetone in 1 M KOH, c = 0.5 M n-propanol in 1 M KOH, d = 0.5 M i-propanol in 1 M KOH, e = 0.5 M ethanol in 1 M KOH and f = 0.5 M propionic acid in 1 M KOH). Ten CV scans were performed for each case. Only the last CV for each is shown.

A 0.5 M concentration of each target compound with 1 M KOH was prepared, and 10 mL of the thus prepared solution was used as electrolyte. Figure 4 shows the voltammetry response with the Pt||PANI-Au_2_ electrode. Remarkable CV response of n-propanol and i-propanol along with a similar response from ethanol is reported. Although n-propanol and i-propanol both show two oxidation peaks at −0.2 V and +0.2 V, for ethanol there is only one peak around −0.2 V. Propionic acid did not show any characteristic response in KOH, due to larger concentration of KOH leading to neutralization within the electrolyte. For the ester group, it is known that esters hydrolyze in alkaline medium [23]. Hydrolysis of ethyl formate in alkaline medium thus follows the following chemical reaction with moderately fast chemical kinetics.

HCOOC_2_H_5_ + 2OH^−^ → HCOO^−^ + C_2_H_5_O^−^ + H_2_O (1)

The product is a carboxylate salt along with alcohol formed from the alkyl group viz. ethanol with alkoxy anion, which is being oxidized. This product can hereby be electrooxidized based on the previous principle. Similarities between voltammetric patterns of ethyl formate and ethanol are shown in the next section.

### 3.3. Principal Component Analysis

**Figure 5:**
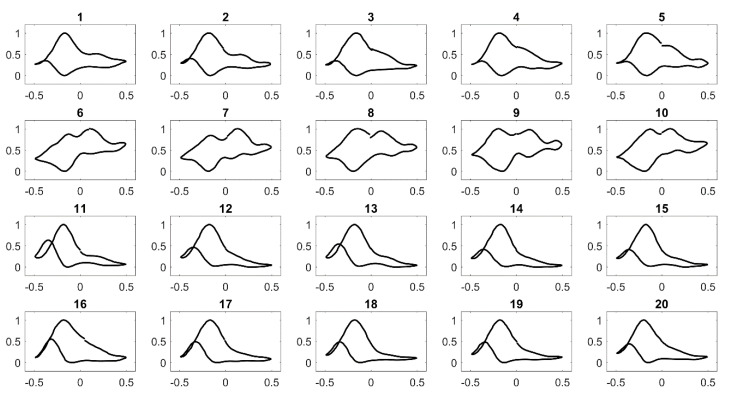
Cyclic voltammograms of the Pt||PANI-Au_2_ electrode vs. Hg/HgO for 4 compounds: (**1**)–(**5**) are CVs of 0.5 M n-propanol in 1 M KOH, (**6**)–(**10**) are CVs of 0.5 M iso-propanol in 1 M KOH, (**11**)–(**15**) are CVs of 0.5 M ethanol in 1 M KOH and (**16**)–(**20**) are CVs of 0.5 M ethyl formate hydrolyzed in 1 M KOH. Ten CV scans were performed for each measurement at scan rate = 100 mV/s. Only the last CV for each measurement is shown.

Using Pt||PANI-Au_2_ as the sensing electrode, a CV dataset was generated by measuring the voltammetric response 5 times on the same electrode for 4 aqueous samples of target compounds—n-propanol, i-propanol, ethanol and ethyl formate (Figure 5). Results showed a good reproducibility in shape of CV for each sample. Furthermore, it was interesting to obtain a large feature set of dimensions and perform classification. Therefore, since the voltammogram has two scanning directions—forward and backward, multidimensional data were extracted by rescaling the CVs between 0 and 1 and slicing data points at intervals of 0.1 V. From a single CV, a 21-dimension feature set (11 data points mined in the forward scan; 10 data points in the backward scan) was then mined each for the 4 samples for 5 measurements [24]. The final feature set size from measurement data shown in Figure 5 was 420 (from 21 dimensions × 5 samples × 4 target compounds). 

**Figure 6:**
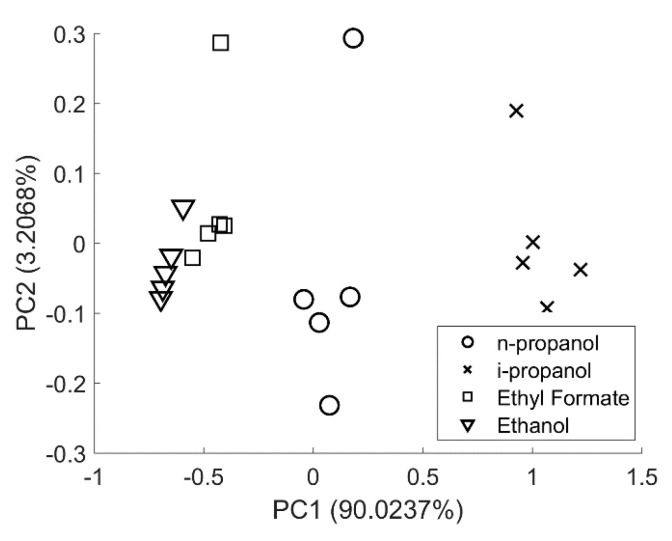
Principal component analysis of multidimensional data obtained from forward and backward scans of cyclic voltammograms of aq. 0.5 M n-propanol in 1 M KOH, aq. 0.5 M i-propanol in 1 M KOH, aq. 0.5 M ethyl formate in 1 M KOH and aq. 0.5 M ethanol in 1 M KOH with the Pt||PANI-Au_2_ electrode.

Figure 6 shows the PCA of multidimensional data obtained with the CV feature set. Data were mean-centered, and a covariance matrix was used to obtain the PCA plot. There is a remarkable separation among the 3 groups of target compounds using only principal component 1 (PC1), which captures 90.02% of the total variance. Samples are seen to cluster nearby with one point away from the clusters formed. On closer examination, it was learnt that these far-away points are the first reading of each target compound. The presence of such points is due to a marginal variation in the location of oxidation peaks across samples for a given target compound. On the other hand, the clusters of ethanol and ethyl formate are very close to each other, as also expected from the similarity in their CV shape, because ethyl formate is rapidly converted to the ethoxy anion.

### 3.4. Indirect Sensing of Ethyl Formate

From the results obtained in aqueous alkaline medium (Figure 4 and Figure S2 in Supplementary Material), it was seen that ethyl formate showed discriminable CV response upon hydrolysis in alkaline medium. Thus, ethyl formate in alkaline KOH electrolyte was chosen as a system for gas sensing. At first, a single-component gas delivery system was constructed and calibrated using a photoionization detector. Calibration showed a full-scale reading of approximately 1600 ppmv in air. Due to limitations of the current resolution of the laboratory-fabricated potentiostat, three widely separated points of relative concentrations were chosen viz. [C]_r_ = 0%, 30%, 60% and 90% for conducting experiments in the gas phase. To dissolve ethyl formate of a desired concentration into the electrolyte (volume = 10 mL), a bubbler system was used. The vapor for bubbling was generated by a gas delivery system based on solenoid values [21]. Ethyl formate vapors at relative concentrations [C]_r_ = 0%, 30%, 60% and 90% were bubbled into four different electrolyte cells containing 20 mL 1 M KOH, each for a period of 300 s. Typical commercial gas sensors have a measurement time of about 240 seconds. Therefore, the selected bubbling time was comparable for visualizing the response. 

**Figure 7:**
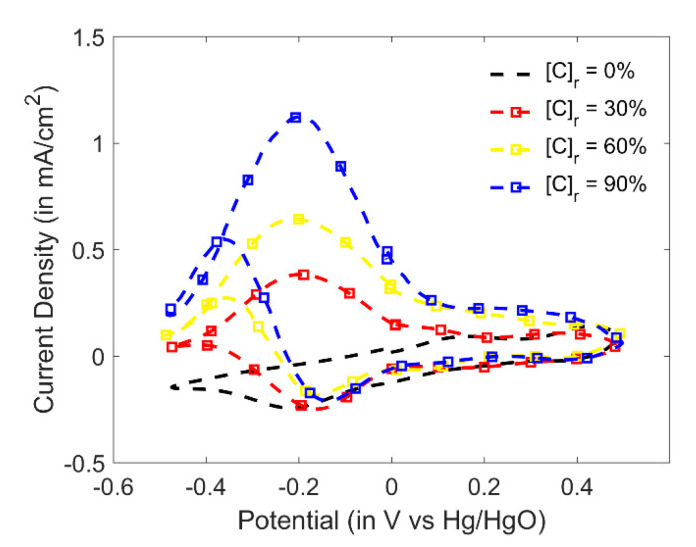
Cyclic voltammograms of the Pt||PANI-Au_2_ electrode vs. Hg/HgO for increasing concentration of gaseous ethyl formate inside the 1 M KOH alkaline medium electrolyte (scan rate = 100 mV/s).

**Figure 8:**
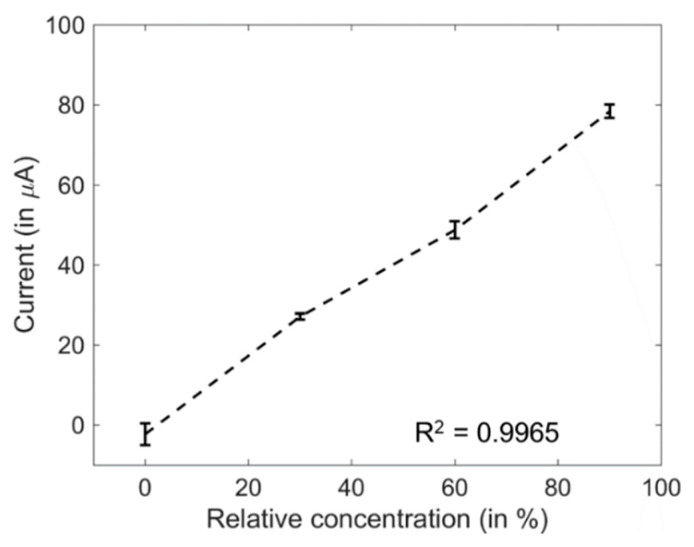
Correlation plot of oxidation peak current at −0.2 V vs. relative concentration of alkaline-hydrolyzed gaseous ethyl formate.

Figure 7 and Figure 8 show the CV responses of ethyl formate in the gas phase in 1 M KOH and the correlation between the relative concentration and the oxidation peak current. The Pt||PANI-Au_2_ electrode yielded a remarkable correlation between the relative concentration generated from the bubbler and the current from the oxidation peak. Furthermore, the CV responses were identical to those obtained by the electrooxidation of ethanol in KOH.

## 4. Discussion

Precisely controlled atomic gold clusters have been formed in the PANI matrix. For this study, bi-atomic gold decorated on a PANI matrix was used as sensing material on a Pt electrode (Pt||PANI-Au_2_), due to its pronounced catalytic activity as compared to single atom decoration. Principal component analysis of multidimensional data from voltammetric response showed classification between three groups of target compounds. Although n-propanol and i-propanol were well-separated, clusters formed from ethyl formate and ethanol overlapped with each other forming a different group. Results obtained in the gas phase operation show that ethyl formate vapor sensing is indirectly possible by electrooxidizing the hydrolysis product. From the coefficient of determination (R^2^), it may be summarized that current characteristics are linear with the output of the gas delivery system used for this study. However, for discrimination between ethyl formate and ethanol, a combination with other gas sensors selective to ethanol is suggested. Furthermore, the detection limit of the order of a few tens of ppm is substantially large when compared with other amperometric gas sensors, where the detection limit is in sub-ppm level [25]. Sensor miniaturization with interdigitated electrodes is suggested since the current density is expected to increase. So far, this study has been limited to C_3_ or compounds with three carbon atoms. In the future, further comparative study is needed for longer carbon chain compounds. It could be interesting to see if the catalytic activity changes by using substrates other than Pt. Electrochemical sensing arrays can then be realized with several combinations of PANI electrodes, with different sizes of atomic clusters on different substrates. Such sensing arrays can be useful for online mixture quantification of odorant compounds and gases [26].

## 5. Conclusions

A bi-atomic Au decorated PANI electrode was used as sensing electrode for ketones, alcohols, esters, and carboxylic acids. Voltammograms of electrooxidation were measured in alkaline medium. Detection of the ethyl formate ester after its hydrolysis in alkaline medium suggests the possibility of indirect sensing. The combination was studied as a gas sensor and linear variations in oxidation peaks were seen from the voltammograms.

## Figures and Tables

**Table 1:** List of target compounds.

S. No.	Target Compound	Chemical Formula	Functional Group	Odor
1.	acetone	C_3_H_6_O	ketone	pungent, cucumber-like
2.	normal-propanol	C₃H₈O	alcohol	mild, alcohol-like
3.	iso-propanol	C₃H₈O	alcohol	strong, alcohol-like
4.	ethanol	C_2_H_6_O	alcohol	strong, alcohol-like
5.	propionic acid	C_3_H_6_O_2_	carboxylic acid	pungent, rancid

## Supplementary Materials

The following are available online at https://www.mdpi.com/1424-8220/20/13/3640/s1, Figure S1: CV of PANI in 0.1 M HClO_4_ at different stages of atomic gold deposition; Figure S2: Cyclic voltammograms of PANI and PANI-Au_2_ for 0.5 M ethyl formate in 1 M KOH (scan rate = 100 mV/sec).
