# Supplementary material for: Indirect Sensing of Lower Aliphatic Ester Using Atomic Gold Decorated Polyaniline Electrode
Source: Sensors (Basel). 2020 Jun 29;20(13):3640. doi: 10.3390/s20133640 (PMC7374289; doi:10.3390/s20133640)
Supplement: Supplementary file 1 [file sensors-20-03640-s001.pdf]

sensors

MDPI

Article

Indirect Sensing of Lower Aliphatic Ester Using Atomic Gold Decorated Polyaniline Electrode

Parthojit Chakraborty [1], Yu-An Chien [2], Tso-Fu Mark Chang [3], Masato Sone [3] and Takamichi Nakamoto [4,*]

1 Department of Information and Communications Engineering, Tokyo Institute of Technology, Kanagawa 226-8503, Japan; chakraborty.p.aa@m.titech.ac.jp
2 Department of Material Science and Engineering, Tokyo Institute of Technology, Kanagawa 226-8503, Japan; chien.y.aa@m.titech.ac.jp
3 Institute of Innovative Research (IIR), Tokyo Institute of Technology, Kanagawa 226-8503, Japan; chang.m.aa@m.titech.ac.jp (T.-F.M.C.); sone.m.aa@m.titech.ac.jp (M.S.)
4 Laboratory for Future Interdisciplinary Research of Science and Technology, Institute of Innovative Research, Tokyo Institute of Technology, Kanagawa 22-68503, Japan
* Correspondence: nakamoto.t.ab@m.titech.ac.jp; Tel.: +81-045-924-5017

Received: 20 May 2020; Accepted: 25 June 2020; Published: 29 June 2020

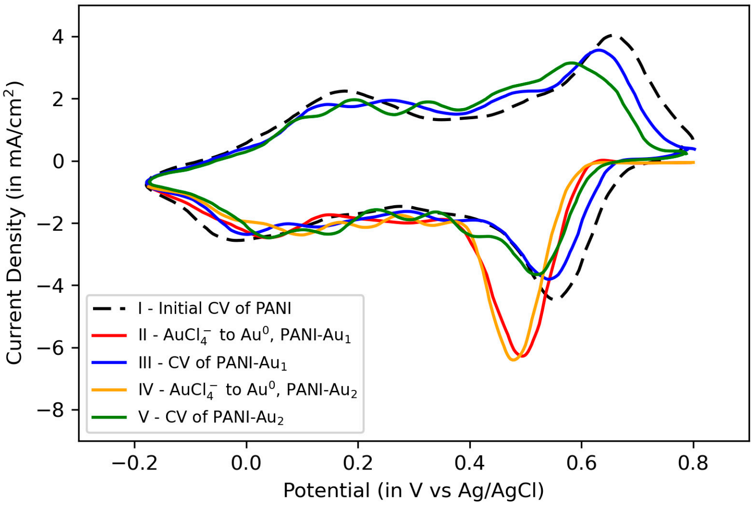

Figure S1. CV of PANI in 0.1 M $HClO_4$ at different stages of atomic gold deposition, (I) Initial CV of PANI before deposition, (II) Reduction of $AuCl_4^-$ to Au^0, formation of PANI-Au_1, (III) CV of PANI-Au_1, (IV) Reduction of $AuCl_4^-$ to Au^0, formation of PANI-Au_2, and (V) CV of PANI-Au_2 (scan rate = 20 mV/s).

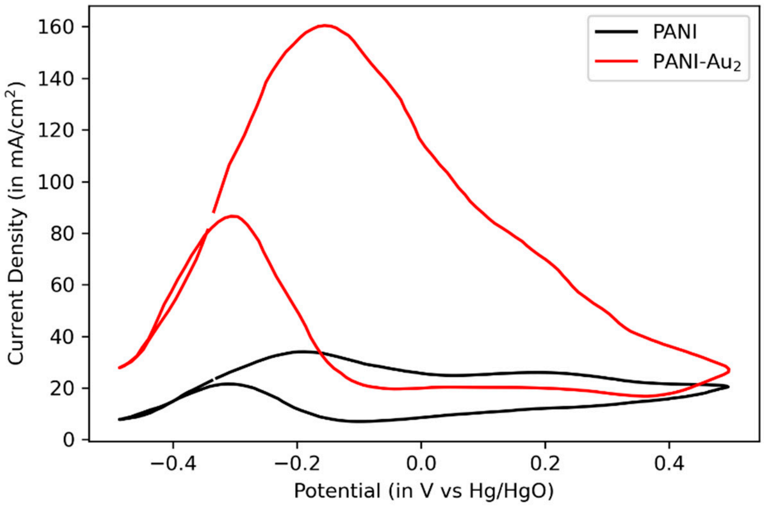

Figure S2. Cyclic voltammograms of PANI and PANI-Au_2 vs. Hg/HgO for 0.5 M ethyl formate dissolved in 1 M KOH (scan rate = 100 mV/sec). Figure shows electrocatalytic activity of bi-atomic Au.

© 2020 by the authors. Licensee MDPI, Basel, Switzerland. This article is an open access article distributed under the terms and conditions of the Creative Commons Attribution (CC BY) license (http://creativecommons.org/licenses/by/4.0/).
